# COVID-19 in a patient with advanced Merkel cell carcinoma receiving immunotherapy

**DOI:** 10.2217/imt-2020-0193

**Published:** 2020-09-09

**Authors:** Cesar Martins da Costa, Zenaide Silva de Souza, Alessandra Corte Real Salgues, Guilherme Harada, Pedro Paulo Marino Rodrigues Ayres, Daniela Bulhões Vieira Nunes, Artur Katz, Rodrigo Ramella Munhoz

**Affiliations:** ^1^Oncology Center, Hospital Sírio-Libanês (HSL), São Paulo, SP, Brazil; ^2^Intensive Care Medicine, Hospital Sírio-Libanês (HSL), São Paulo, SP, Brazil

**Keywords:** anti-PD-1 therapy, coronavirus, COVID-19, immune-related adverse events, immunotherapy, Merkel cell carcinoma, oncology, pandemic, SARS-CoV-2, skin cancer

## Abstract

**Background:** Little is known about the 2019 novel coronavirus disease (COVID-19) course and outcomes in patients receiving immunotherapy. Here we describe a metastatic Merkel cell carcinoma patient with a severe acute respiratory syndrome coronavirus 2 infection while receiving pembrolizumab. **Case presentation:** A 66-year-old man, with a metastatic Merkel cell carcinoma receiving pembrolizumab, presented with fever. Chest computed tomography (CT) showed pulmonary ground-glass opacities, suggesting viral or immuno-related etiology. On day 7, the patient was hospitalized due to dyspnea and worsening of the radiological findings. Real time polymerase chain reaction (RT-PCR) testing confirmed COVID-19. The patient developed acute respiratory distress syndrome and acute kidney injury. Hydroxychloroquine was administered for 5 days, but discontinued after supraventricular extrasystoles. Clinical improvement allowed the patient’s discharge after 81 days of hospitalization. **Conclusion:** A careful evaluation of oncologic patients receiving immunotherapy during the COVID-19 pandemic is of utmost importance.

The 2019 novel coronavirus disease (COVID-19), caused by the severe acute respiratory syndrome coronavirus 2 (SARS-CoV-2), emerged in Wuhan city and rapidly spread throughout China, causing a wide spectrum of severity and being classified by the WHO as a public health emergency of international concern. To date, WHO estimates that this pandemic has affected 20 million people and taken the lives of more than 750,000 individuals worldwide, not including the unreported cases; and its reach and severity continue to spread [1].

Despite the alarming numbers, the burden of this disease and clinical course has been poorly characterized in cancer patients. Initial reports suggested an increased risk for severe forms of COVID-19 in this population [2,3], posing a major concern for oncologists and healthcare providers in this new scenario. Susceptibility to infections is greater in individuals with cancer because of their immunosuppressive state related to the tumor and oncological treatments (chemotherapy, surgery and radiotherapy) [2,3]. A Chinese study has analyzed 18 cancer patients and compared them with 1572 patients without cancer. After adjusting for age, comorbidities and smoking history, cancer was associated with a higher risk of severe events, defined as intensive care unit (ICU) admission requiring invasive ventilation, or death (OR: 5.4; 95% CI: 1.80–16.18; p = 0.0026); in addition, cancer patients presented severe events earlier than patients without cancer (median time 13 vs 43 days, respectively; HR: 3.56; 95% CI: 1.65–7.69; p < 0.0001) [3].

Another area of uncertainty is whether patients receiving different types of antitumor treatments (e.g., chemotherapy, target-therapy or immune checkpoint inhibitors [ICI]) are subject to distinct risks and outcomes of COVID-19. As an example, the use of ICI such as anti-programmed death 1/ligand-1 (anti-PD-1/PD-L1) and anti-cytotoxic T-lymphocyte antigen 4 (anti-CTLA-4) monoclonal antibodies has led to a new understanding of oncology [4], but many questions about immunomodulation process are still an area of intense research [5,6] and the role of ICI as a risk or protective factor in SARS-Cov-2 infection has yet to be elucidated. In this setting, the differential diagnosis between ICI-induced pneumonitis and COVID-19 can be a challenge, since both may share similarities in clinical presentation and overlapping radiological findings, but distinct therapeutic approaches [7,8]. Here we describe the successful management of a patient with metastatic Merkel cell carcinoma that developed a severe SARS-CoV-2 infection while receiving pembrolizumab.

## Case presentation

**Figure 1.**
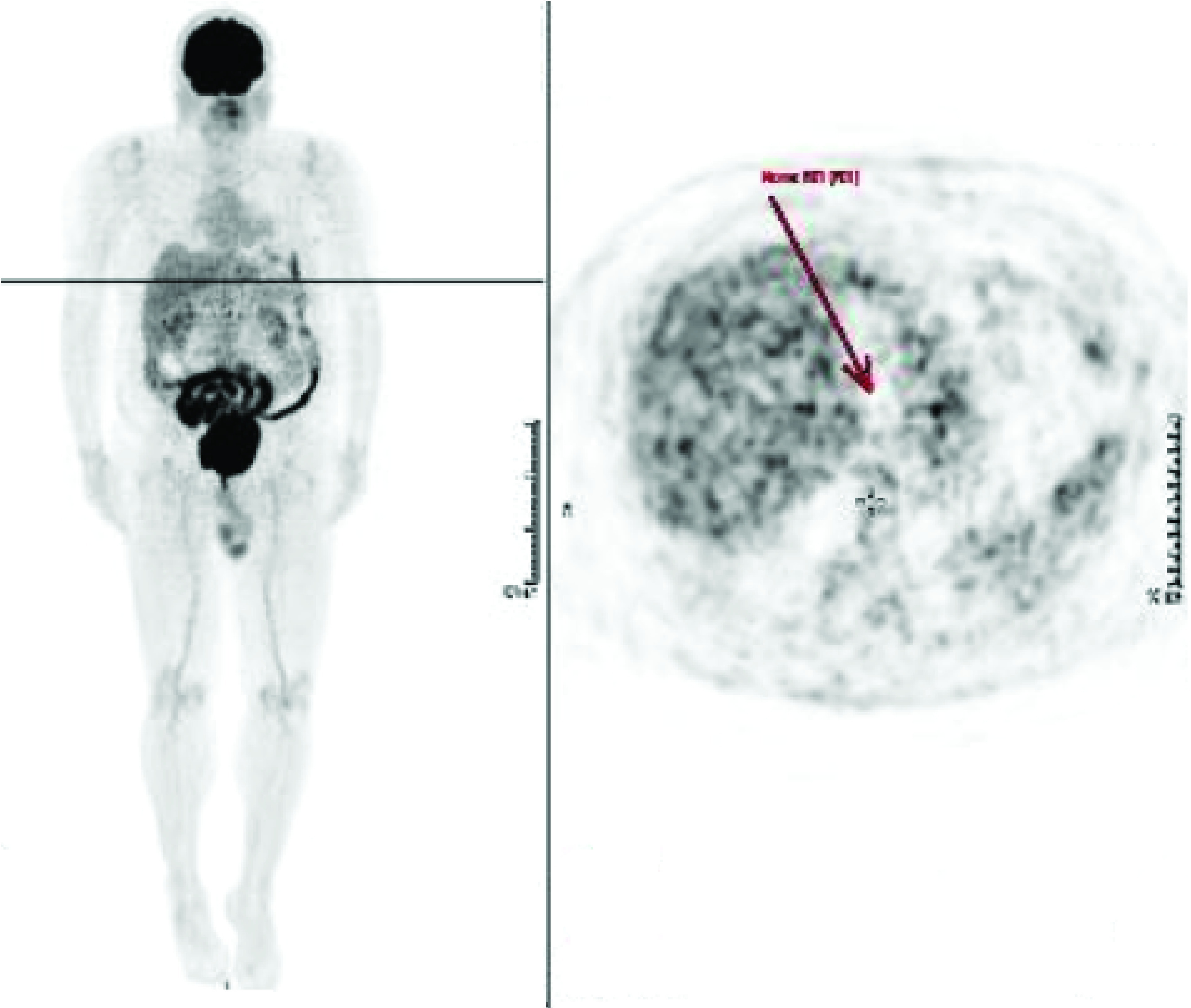
**PET-CT scan.** Metabolic and morphological complete resolution of the left perigastric lymph node, indicating complete response to treatment.

A 66-year-old man, former smoker, with a history of hypertension and diabetes, was diagnosed in 2018 with a Merkel cell carcinoma (MCC) of the gluteal region measuring 3.0 cm in the greatest diameter (pathological staging: pT2pN0, stage IIA) that was treated with surgery and adjuvant radiation therapy. In February 2019, a distant tumor relapse in a perigastric lymph node was confirmed. The patient started systemic therapy with avelumab 10 mg/kg every 14 days, however, he developed recurrent infusion reactions despite the use of prophylactic premedications, increasing in severity until the third cycle, which led to a substitution for pembrolizumab 200 mg every 3 weeks. The patient reached a complete metabolic response in November 2019 (Figure 1).

**Figure 2.**
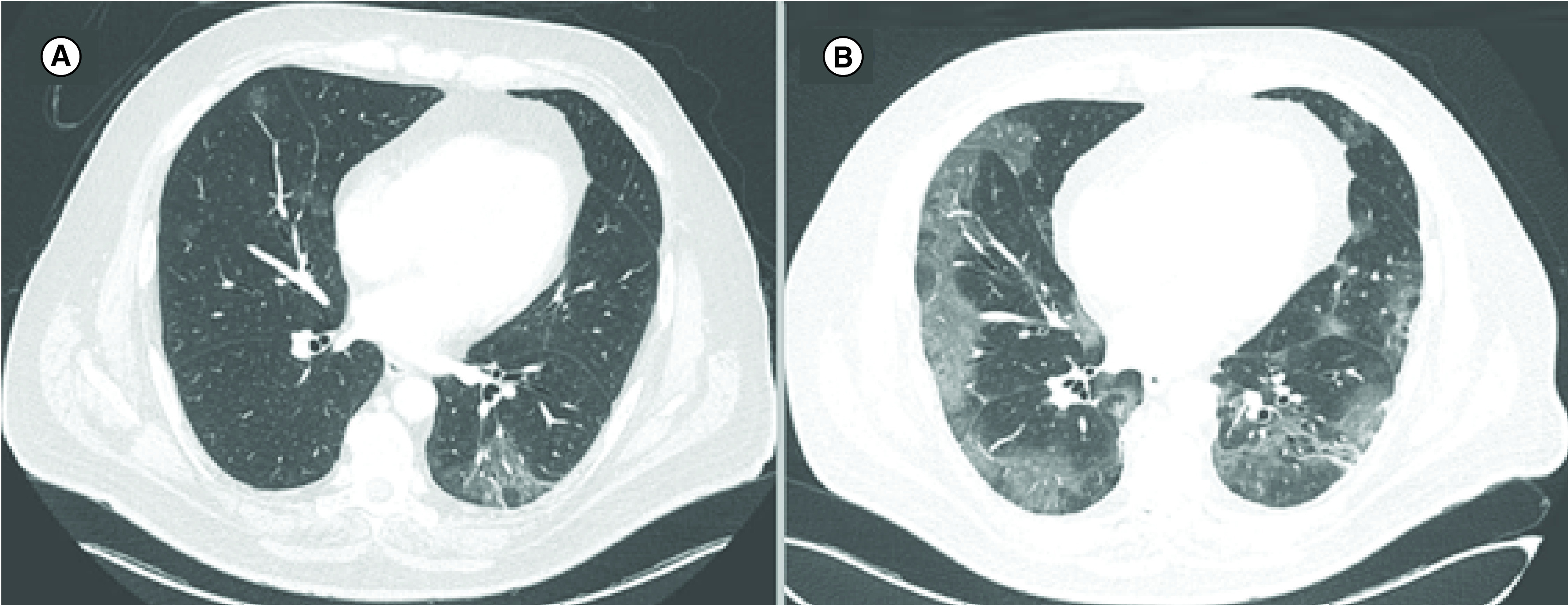
Chest CT. Chest CT showing sparse and bilateral ground-glass opacities, the largest in the left lower lobe, with an inflammatory aspect **(A)**. Deterioration of the ground-glass opacity and bilateral patchy shadowing 7 days later **(B)**.

After 13 cycles of maintenance pembrolizumab (last infusion: 5 March 2020), the patient presented with fever, but no respiratory symptoms initially. Chest CT was performed and showed a bilateral pulmonary inflammatory pattern (day 1), comprising the possibilities of a viral infection or immune-related pneumonitis attributable to pembrolizumab (Figure 2). As the patient was oligosymptomatic and in good general condition, immunotherapy was suspended and home isolation indicated, with daily medical follow-up. COVID-19 test was not performed on outpatients, in compliance with the recommendations of the Brazilian Ministry of Health at that time. On day 7 (23 March 2020), the patient was admitted to the hospital due to the onset of dyspnea. RT-PCR testing was positive for SARS-CoV-2 and chest CT demonstrated a significant worsening of the bilateral pulmonary ground-glass opacities (Figure 2). Despite the initial support, the patient evolved to acute respiratory distress syndrome and developed acute kidney injury on day 9 (Table 1), requiring invasive mechanical ventilation and wide spectrum antibiotics, including azithromycin. Hydroxychloroquine was added on day 12, but discontinued 5 days later due to supraventricular extrasystoles. Although the patient showed improvements in renal function and ventilatory parameters, he maintained impaired consciousness following sedation withdrawal. A head CT and electroencephalogram were obtained, and showed no significant abnormalities. Cerebrospinal fluid examination did not show signs of infection and cerebrospinal fluid RT-PCR testing was negative for SARS-CoV-2. Anticoagulation was started due to a deep vein thrombosis identified in brachial vein on day 20, and on day 25 the patient was submitted to a tracheostomy. He ultimately progressed with ventilatory and neurological improvement, and prolonged rehabilitation, until hospital discharge on 12 June 2020.

**Table 1.** Clinical laboratory results and ventilatory parameters.

	Day 7	Day 9	Day 14	Day 26
Hemoglobin/hematocrit (g/dl and %)	16.1/46.8	14.9/43.8	12.7/40.2	9.6/30.4
Leukocytes (g/dl)	6650	3810	3420	5360
Neutrophils (g/dl)	6300	3350	2810	4650
Lymphocytes (g/dl)	210	270	260	260
Platelets(/mm^3^)	207,000	271,000	262,000	303,000
Creatinine (mg/dl)	1.42	1.00	2.09	1.03
Urea (mg/dl)	81	56	97	90
LDH (U/l)	717	764	415	
CRP (mg/dl)	9.16	18.27	9.85	14.66
PCR Ncov-2019	Positive			
Genexpert influenza A,B e H1N1	Undetectable			
D-dimer		1015	1724	886
**Arterial blood gas analysis**				
pH		7.49	7.37	7.59
pO_2_ (mmHg)		69	67	113
pCO_2_ (mmHg)		29	49	31
HCO_3_ (mmol/l)		22	27	30
SatO_2_ (%)		94	93	98
**Ventilatory parameters**				
FiO_2_ (%)		35	30	25
PEEP		8	10	7

## Discussion

Early studies have suggested that cancer patients have a higher risk of developing COVID-19 than the general population, with an increased chance of serious respiratory complications and prolonged time in the ICU [2,3]. Whether distinct types of cancers and different antitumor treatments confer similar risks for severe SARS-Cov-2, it remains inconclusive, especially in patients receiving immunotherapy.

A retrospective study conducted at the Memorial Sloan Kettering Cancer Center (NY, USA) sought to examine the impact of PD-1 blockade on severity of COVID-19, in which 41 (59%) out of 69 consecutive outpatients with lung cancer infected by SARS-Cov-2 were previously treated with anti-PD-1 therapy. Parameters such as hospitalization (OR: 1.66; 95% CI: 0.6–4.60), ICU/intubation/do not intubate (OR: 1.18; 95% CI: 0.42–3.40) and death (OR: 1.81; 95% CI: 0.57–6.43) were not statistically significant for the association of PD-1 blockade exposure and COVID-19 severity [9]. Despite these results, data from the same institution showed different conclusions: in a multivariate analysis of 423 cancer patients, ICI treatment was a predictor of severe respiratory illness (HR: 2.74; 95% CI: 1.37–5.46; p = 0.004) and also a predictor of hospitalization for COVID-19 (OR: 2.84; 95% CI: 1.24–6.72; p = 0.013) [10]. In an effort to understand the risk factors for morbidity and mortality from this novel virus, a global consortium (TERAVOLT) recently reported data from eight countries including a total of 200 patients with COVID-19 and thoracic cancers. Chemotherapy–immunotherapy combination and ICI alone were performed in 14 and 23%, respectively; but they did not impact on risk of death (OR: 0.989; 95% CI: 0.276–3.183 for chemotherapy–immunotherapy and OR: 1.385; 95% CI: 0.524–3.639 for ICI alone) [11]. All these results should be interpreted with caution due to the limitations of each study, such as small sample size, retrospective analysis, short prospective follow-up, selected population, pre-existing individual diseases, recent or previous smoking and other methodological bias.

Furthermore, the relationship of immunomodulation and severity of SARS-Cov-2 infection was also correlated with NK and T-cell exhaustion during the immune response, which may result in viral disease progression and rapid clinical decompensation. In a cohort of 68 COVID-19 patients (55 with mild disease [MD] and 13 with severe disease [SD]), the total NK and CD8^+^ T cells counts by peripheral blood samples were collected on admission at the Anhui Medical University in China. Patients with SD have significantly lower number of T cells, CD8^+^ T cell and NK cells counts compared with those with MD (p < 0.05) and 25 healthy controls (p < 0.0001), indicating an innate and adaptive exhaustion related to SARS-Cov-2 infection [12]. These results are in accordance with the laboratorial findings of Guan *et al.*, which reported lymphocytopenia related to COVID-19 in 83.2% of the patients on hospital admission [13]. Exhausted T cells upregulate selectively PD-1 receptors, and anti-PD-1 blockade can restore T CD8^+^ functions such as proliferation, cytokines secretion and cell death. The use of anti-PD-1 therapy has been associated with an increased cellular immunity in cases of viral, bacterial and fungal infections [14], also decreasing viral load *in vivo* [15]. However, overactivation of T cells contributes to a severe immune injury, leading to COVID-19 related acute respiratory distress syndrome [16]. Therefore, the use of immunotherapy could potentially affect the response and course of a viral infection, although the role of anti-PD-1 therapy and COVID-19 severity is not yet established in prospective randomized trials.

In addition, the clinical and radiological similarities between COVID-19 and ICI-induced pneumonitis may represent a pitfall in clinical practice. Pneumonitis as an immune-related adverse event (irAE) affects almost 5% of patients receiving anti-PD-1/PD-L1 in monotherapy and approximately 10% in anti-CTLA-4/anti-PD-1 combination therapy [17], rarely leading to death [18]. Radiological features of ICI-induced pneumonitis are non specific, including parenchymal consolidations, interstitial pneumonia and peribronchial or peripheral ground-glass opacities [19]. The latter is the most common radiological finding related to SARS-Cov-2, accounting for 56.4–75.0% of cases [13,20], and the former (46.3%) is related to a higher risk for developing severe COVID-19 events (HR: 5.438; 95% CI: 1.498–19.748; p = 0.010) [20]. COVID-19 pneumonia mimicking ICI-induced pneumonitis was also discussed by Artigas *et al.*, reporting that hypermetabolic ground glass opacities with peripheral distribution by 18F-FDG PET/CT may appear even in SARS-Cov-2 infection or irAE induced by anti-PD-1 therapy [21]. Therefore, differential diagnosis between irAEs and SARS-Cov-2 infection always deserves a dedicated workup.

Guidelines have been published to guide and allow safe patient care, without jeopardizing the ongoing oncological treatment [22], including recommendations for skin cancer management [23]. In this context, preventive measures must be implemented in oncology departments, in order to minimize patient exposure to COVID-19. Early diagnosis of SARS-Cov-2 with RT-PCR is essential, even in asymptomatic patients, preventing nosocomial spread in cancer clinics [24]. Shared decision making with physicians and patients about delays in the oncologic treatment must occur, considering an individual’s risk of COVID-19 complications against the worse prognosis from postponed cancer therapy [23]. Retrospective data suggest that chemo–immunotherapy can be safely delivered even in this pandemic scenario, but sometimes may require regimen modifications or alternative schedules that should also be determined on an individual basis [25].

## Conclusion

Testing for the novel coronavirus is a top priority in response to the global SARS-CoV-2 pandemic, avoiding equivocal diagnosis or treatment delays. A careful evaluation and a rapid assessment of oncologic patients receiving ICI during the COVID-19 pandemic are essential. Gathering evidences to better characterize the spectrum of manifestations of COVID-19 and to guide tailored management algorithms in patients receiving ICI remains of utmost importance.

**Summary points**

- The 2019 novel coronavirus disease (COVID-19) is a public health emergency of international concern.
- The role of immune checkpoint inhibitors in severe acute respiratory syndrome coronavirus 2 (SARS-Cov-2) infection has not been established yet in prospective trials, although retrospective data suggest that chemo-immunotherapy can be safely delivered, eventually requiring regimen modifications or alternative schedules determined on an individual basis.
- NK cells and T lymphocytes exhaustion during the immune response against the SARS-Cov-2 infection is associated with PD-1 upregulation, viral disease progression and clinical decompensation.
- Anti-PD-1 blockade can restore T CD8^+^ functions, which improves cellular immunity.
- Immune overactivation, however, can lead to acute respiratory distress syndrome related to COVID-19.
- Suspected SARS-Cov-2 infection may mimic immune-related adverse events in those patients receiving immunotherapy, especially pneumonitis, which deserves a dedicated workup.
- Testing for the novel coronavirus is a top priority in response to the global SARS-CoV-2 pandemic, avoiding equivocal diagnosis or treatment delays.
- Evidences are needed to guide tailored management algorithms in patients receiving immune checkpoint inhibitor during the COVID-19 pandemic.
